# Supplementary material for: Artificial Intelligence-Based Prognostic Model for Urologic Cancers: A SEER-Based Study
Source: Cancers (Basel). 2022 Jun 26;14(13):3135. doi: 10.3390/cancers14133135 (PMC9264864; doi:10.3390/cancers14133135)
Supplement: Supplementary file 1 [file cancers-14-03135-s001.zip › cancers-1758834-SI.pdf]

Supplementary Materials: Artificial Intelligence-based Prognostic Model for Urologic Cancers: A SEER-based Study

Okyaz Eminaga, Eugene Shkolyar, Bernhard Breil, Axel Semjonow, Martin Boegemann, Lei Xing, Ilker Tinay, and Joseph C. Liao

Table of Contents

Supplementary figure S1
Supplementary file S1
Dataset
Definition of development and out-held test sets
Feature selection
Hyperparameter configuration for the model development
Model calibration for the cancer-specific mortality risk estimation
References

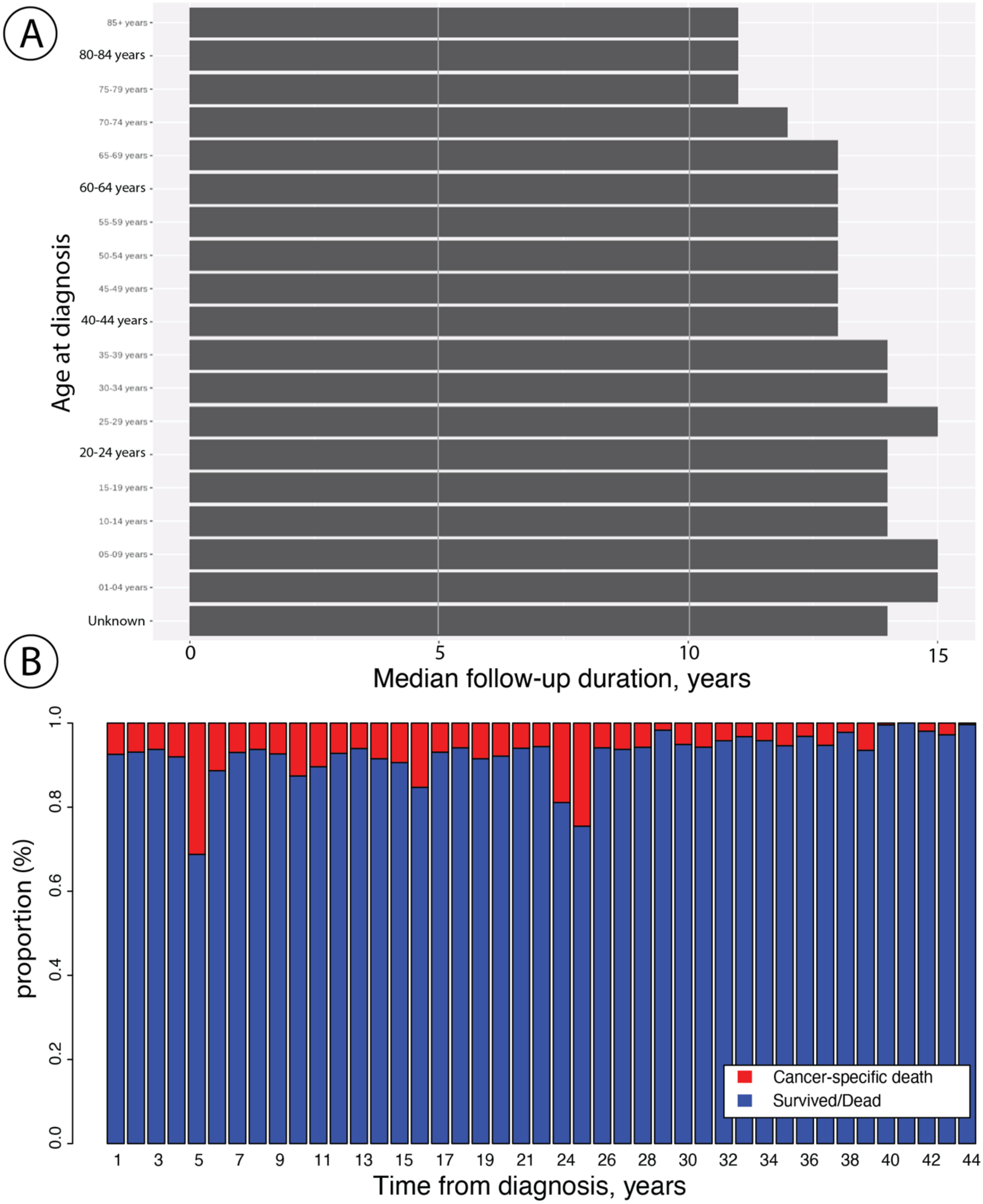

Figure S1: (A) The median follow-up duration in years after stratifying by age at diagnosis (categorized), showing a decline in the median follow-up duration with aging. (B) the relative frequencies that alter over the years. Overall, the cancer-related death distribution has a declining trend with time. “85+”: the patient age is equal to or above 85 years.

Supplementary File S1

Dataset

We utilized the SEER database covering the years 1975-2017 (Version 18) and included 2,006,052 patients diagnosed with one of the urologic cancers (i.e., prostate, testis, kidney, urinary bladder, ureter, renal pelvis, penis, other genital organs). The affected organ by cancer type was determined according to the documentation guideline of the SEER program (Site recode B ICD-O 3/WHO 2008). A total of 505 features from the database were available and covered demographic, clinical and pathological information. Information on treatments was not included, since only surgery data are available. Furthermore, two additional features (cancer-specific death status and the follow-up duration given in years) included the cancer-specific survival follow-up data. Cases in which patients died due to the primary cancer in urogenital organs were defined as cancer-specific death. The follow up duration was determined based on the last contact with cancer survivors or the cancer-specific death of the patients.

Definition of development and out-held test sets

The validation of the model generalization ideally requires a test set that represents a considerable portion of the population. The SEER database covers the most variations in the disease conditions and characteristics compared to any data from single or two institutions [1] and represents approximately 30% of the U.S. population [2]. We preferred the conventional validation approach due to the fact that the SEER database is representative of the U.S. population; any local dataset for external validation would not provide the same representation of the population as the SEER database.

We randomly divided the database into the development set (90%) and the out-held test set (10%), while maintaining the same proportion for the cancer-specific death status cases between these datasets for model development and evaluation. The development and test sets were disjointed to ensure no case overlapped between the data sets. Therefore, the out-held test derived from the SEER database met the requirements and validated the generalization of our approach. The development set was then split into the training (90%) and validation sets (10%). The validation set was used to evaluate the optimization procedure of the model weights. We applied feature-wise normalization by ranking the units of a feature according to appearance order in the dataset and dividing by the maximum rank to achieve a value between 0 and 1. Where the information was missing for the feature, a value of -1 was given. In contrast, the follow-up duration was fed as a non-negative continuous value without normalization into the model, and the cancer-specific death status was binarized (0: no cancer-specific death; 1: cancer-specific death). The reason for considering cancer-specific death status instead of overall survival (OS) was to mitigate any potential biases associated with non-cancer specific survival during the model development.

Feature selection

According to good practice in data science, data preprocessing is essential to achieve a good model. Furthermore, any model used for clinical decision making should incorporate features that are well-established and accessible during the clinical routine. Therefore, we aimed to select features for survival modeling that had the relevant clinical information, covering tumor stage and biomarkers in addition to age at diagnosis and race due to their well-established clinical importance in urologic cancers [3-7]. Here, we manually selected sixteen clinically relevant features which are well-

documented and currently considered by the SEER program. Furthermore, these features are well-established parameters for clinical decision making, as listed in **Table 1**. While selecting the features, we intended to reduce the version dependency of the tumor stage in our model by considering features that describe the tumor extension and tumor dissemination in more detail. The SEER tumor stage was considered given that the SEER program used this feature to estimate the cancer-specific survival rate according to the tumor dissemination status. These features were selected with the full agreement of five urologists (OE, AS, MB, JL, IT) with more than 10 years of profound clinical and research experience in urologic cancers.

Table S1. lists the features selected for survival modeling. ** denotes that this feature was derived from cancer-specific features. ++ the definition of the features varies by the information sources for staging (e.g., biopsy or surgery). Due to the variational options for cancer-specific features, we only provided the feature summary. Details regarding the available options can be obtained from https://seer.cancer.gov.info.CS: Cancer-specific; the number of CS site-specific factor is defined by the SEER program to differentiate between different CS site specific factors.

Feature	Input data.
**Common features**	
Age at diagnosis	Continuous value ranged between 18 and 114 years
Race	White<br>Black<br>Others<br>Unknown
Total number of malignant tumors for the patient	Continuous value ranged between 1 and 10
Regional nodes positive 1988	The number of positive lymph nodes:<br>The range between 0 and 90<br>>=90<br>Unknown or not performed (-1)
Regional nodes examined 1988	The number of lymph nodes examined:<br>The range between 0 and 90<br>>=90<br>Unknown or not performed (-1)
SEER historic stage A **	Tumor stage summary:<br>Localized

	Distant<br>Regional<br>Localized and/or regional (Prostate Cancer)					
Time, year	The follow-up duration until the last check or death.					
**Cancer-specific features (n)**	**Prostate Cancer (9)**	**Kidney Cancer (5)**	**Urinary Bladder Cancer (5)**	**Renal Pelvis or Ureter Cancer (5)**	**Testicular Cancer (5)**	**Penile Cancer (4)**
CS site-specific factor 2	The measurement result of serum PSA level (ng/mL)	The vein involvement (Vena Cava and vena renalis)	The length of the maximum lymph node metastasis		Not applicable	
CS Mets at dx 2004	Evidence of metastases at the time of diagnosis.:<br>The definition varies between cancers of the genitourinary system. Detailed information for the cancer-specific list can be obtained from the SEER program.					
Derived AJCC M 6th ed 2004**	M0<br>MX<br>M1<br>M1a<br>M1b<br>M1c	M0<br>M1<br>MX	M0<br>M1<br>MX	M0<br>M1<br>MX	M0<br>M1<br>M1a<br>M1b<br>MX	M0<br>M1<br>MX
CS site-specific factor 12	Number of positive biopsy cores	Not applicable				
CS site-specific factor 13	Number of total biopsy cores	Not applicable			Postoperative Alpha	Not applicable

					Fetoprotein (AFP) level	
CS extension 2004[++]	Tumor stage and extension:<br>The definition varies between cancers of the genitourinary system. Detailed information for the cancer-specific list can be obtained from the SEER program.					
CS site-specific factor 7[++]	First and second Gleason patterns in Biopsy or TURP specimens	Not applicable				
CS site-specific factor 9[++]	First and second Gleason patterns in prostatectomy specimen	Not applicable				
Derived AJCC N 6th ed 2004 (Lymph node metastases status)	N0<br>N1<br>NX	N0<br>N1<br>NX	N0<br>N1<br>N2<br>N3<br>NX	N0<br>N1<br>NX	N0<br>N1<br>N2<br>N3<br>NX	N0<br>N1<br>N2<br>N3<br>NX

Hyperparameter configuration for the model development

A grid search was applied to identify the optimal hyperparameter configuration for the model architecture of the recurrent neural network (i.e., simple recurrent neural network (sRNN), gated recurrent units (GRU) [8] and Long Short-Term Memory unit (LSTM) [9]) and model optimization algorithms with the default configuration (i.e., Adaptive Moment Estimation (Adam) [10] and Root Mean Square Propagation (RMSprop) [11]). The loglikelihood function was measured to evaluate the goodness of fit of these models with a predefined decay learning rate (start learning rate: 0.001 and decay rate: 0.8, after every fifth epoch). Furthermore, we evaluated the inclusion of the dropout in the model on the loglikelihood. The linear regression function was applied for the final activation function and the mean square error was used as a loss function. The lowest value for the loglikelihood on the in-training validation set indicated the best hyperparameter configuration. Given the explorative character of the hyperparameter study, utilizing a subset of the development set (n=10,000) was adequate to determine the optimal hyperparameter configuration. Each model with different hyperparameter configuration was trained for 10 epochs to limit the computational time

consumption. When the optimal hyperparameters configuration was identified, the final model with this configuration was trained on the whole training set until convergence.

Model calibration for the cancer-specific mortality risk estimation

We utilized the Kaplan–Meier (KM) estimates to calibrate the trained model for cancer-specific survival estimates on the training set and tested the model fitness on the test set. For the model calibration, we utilized the lowest and highest boundaries for the 95% confidence intervals of the survival estimates for a follow-up period ranging from 1 years to 44 years. Then, we applied Brent's optimization algorithm [12] to determine the residual ($\alpha \epsilon$ [0,1]), with the minimum for the equation calculating the non-negative differences (Δ) between the true KM survival estimates $f(y)$ and predicted KM survival estimates $f(\hat{y} + \alpha)$.

$$\Delta = [f(y) - f(\hat{y} + \alpha)]^2 \ where \ \Delta \in [0,1]$$

A difference of 0 ($\Delta = 0$) means a perfect fitness between the true and predicted KM estimates, while a difference of 1 ($\Delta = 1$) means poor model calibration for the survival estimation. After incorporating the residual into the prediction model, the fitness of the model was assessed by comparing KM Curves between the prognosticated and observed probabilities and by applying the previous equation on the out-held test set [13].

References

1. Eminaga, O.; Al-Hamad, O.; Boegemann, M.; Breil, B.; Semjonow, A. Combination possibility and deep learning model as clinical decision-aided approach for prostate cancer. *Heal. Informatics J.* **2019**, *26*, 945–962
2. Duggan MA, Anderson WF, Altekruse S, Penberthy L, Sherman ME. The Surveillance, Ep-idemiology, and End Results (SEER) Program and Pathology: Toward Strengthening the Critical Relationship. Am J Surg Pathol. 2016;40, e94-e102.
3. Babjuk, M.; Böhle, A.; Burger, M.; Capoun, O.; Cohen, D.; Compérat, E.M.; Hernández, V.; Kaasinen, E.; Palou, J.; Rouprêt, M.; et al. EAU Guidelines on Non–Muscle-invasive Urothelial Carcinoma of the Bladder: Update 2016. *Eur. Urol.* **2016**, *71*, 447–461
4. Ljungberg, B.; Albiges, L.; Abu-Ghanem, Y.; Bensalah, K.; Dabestani, S.; Fernandez-Pello, S.; Giles, R.H.; Hofmann, F.; Hora, M.; Kuczyk, M.A.; et al. European Association of Urology Guidelines on Renal Cell Carcinoma: The 2019 Update. *Eur Urol* **2019**, *75*, 799-810.
5. Roupret, M.; Babjuk, M.; Comperat, E.; Zigeuner, R.; Sylvester, R.; Burger, M.; Cowan, N.; Bohle, A.; Van Rhijn, B.W.; Kaasinen, E.; et al. European guidelines on upper tract urothelial carcinomas: 2013 update. *Eur Urol* **2013**, *63*, 1059-1071.
6. Witjes, J.A.; Lebret, T.; Compérat, E.M.; Cowan, N.C.; De Santis, M.; Bruins, H.M.; Hernández, V.; Espinos, E.L.; Dunn, J.; Rouanne, M.; et al. Updated 2016 EAU Guidelines on Muscle-invasive and Metastatic Bladder Cancer. *Eur. Urol.* **2016**, *71*, 462–475
7. Stephenson, A.; Eggener, S.E.; Bass E.B.; Chelnick D.M.; Daneshmand, S.; Feldman, D.; Gilligan, T.; Karam, J.A.; Leibovich, B.; Liauw, S.L.; Masterson, T.A.; Meeks, J.J.; Pierorazio, P.M.; Sharma, R.; Sheinfeld, J. Diagnosis and treatment of early stage testicular cancer: AUA guideline. *The Journal of urology.* **2019**, *202*, 272–281.
8. Chung J, Gulcehre C, Cho K, Bengio Y. Empirical evaluation of gated recurrent neural networks on sequence modeling. *arXiv preprint arXiv:14123555.* **2014**.
9. Hochreiter, S.; Schmidhuber, J. Long short-term memory. *Neural computation.* **1997**, *9*, 1735–1780.
10. Kingma DP, Ba J. Adam: A method for stochastic optimization. *arXiv preprint arXiv:14126980.* **2014**.
11. Hinton G, Srivastava N, Swersky K. Neural networks for machine learning lecture 6a overview of mini-batch gradient descent.
12. Brent RP. *Algorithms for minimization without derivatives.* Courier Corporation; 2013.
13. Royston, P.; Altman, D.G. External validation of a Cox prognostic model: principles and methods. *BMC Med Res. Methodol.* **2013**, *13*, 33.
